# Dietary patterns influence the in silico GABA production capacity of *Bifidobacterium adolescentis* HD17T2H and other human gut bacteria

**DOI:** 10.1038/s41598-026-43006-9

**Published:** 2026-03-11

**Authors:** Ann Homscheid, Karlis Arturs Moors, Bram Nap, Wolfgang Lieb, Andre Franke, Matthias Laudes, Ines Thiele, Christoph Kaleta, Georgios Marinos

**Affiliations:** 1 Research Group Medical Systems Biology, Institute of Experimental Medicine, University Hospital Schleswig-Holstein Campus Kiel, Kiel University, Kiel, Schleswig-Holstein, Germany; 2 CAU Innovation GmbH, Kiel University, Kiel, Schleswig-Holstein, Germany; 3 School of Medicine, University of Galway, Galway, Ireland; 4 Ryan Institute, University of Galway, Galway, Ireland; 5 Digital Metabolic Twin Centre, University of Galway, Galway, Ireland; 6 Institute of Epidemiology, University Hospital Schleswig-Holstein Campus Kiel, Kiel University, Kiel, Schleswig-Holstein, Germany; 7 Institute of Clinical Molecular Biology, University Hospital Schleswig-Holstein Campus Kiel, Kiel University, Kiel, Schleswig-Holstein, Germany; 8 Institute of Diabetes and Clinical Metabolic Research, University Hospital Schleswig-Holstein, Campus Kiel, Kiel, Schleswig-Holstein, Germany; 9 Division of Microbiology, University of Galway, Galway, Ireland; 10 APC Microbiome Ireland, Cork, Ireland

**Keywords:** Microbiome, Gut-Brain axis, Genome-scale metabolic modelling, Flux balance analysis, Flux variability analysis, Human nutrition, Human cohort, *Bifidobacterium adolescentis* strain HD17T2H, Gamma-aminobutyric acid, Computational biology and bioinformatics, Microbiology, Neuroscience

## Abstract

Gamma-aminobutyric acid (GABA) is a neurotransmitter that inhibits neuronal excitability and also affects mucosal function and gut motility. Importantly, while the gut microbiome is a known source of GABA, little is known about the production capacities of its specific members. In our study, we investigated in silico how 11 predefined diets influence GABA production by *Bifidobacterium adolescentis* HD17T2H, an important GABA producer among bifidobacteria within the human gut microbiota. We show that the GABA production potential of *B. adolescentis* strain HD17T2H varies considerably across diets, with the vegetarian diet showing the highest potential and the ketogenic diet the lowest. Further, we analysed which specific compounds raised GABA production. Our in silico predictions revealed that carbohydrates and nitrogen-rich compounds, such as amino acids, strongly increase GABA production. We also analysed personalised nutritional data from a human cohort (Kiel cohort), in an in silico approach. In doing so, we found 87 potent GABA-producing strains across 47 bacterial genera, including *Burkholderia*, *Pseudomonas*, and *Delftia*, suggesting that not only commensals but also bacterial pathogens contribute to GABA production. Thus, our modelling approach highlights that nutrient availability is a central determinant of bacterial GABA production.

## Introduction

Gamma-aminobutyric acid (GABA) is a non-proteinogenic amino acid with a range of known physiological and psychological effects. In the mature brain of mammals, GABA mainly functions as an inhibitory neurotransmitter^1^. Changes in GABA receptor expression are associated with anxiety and depressive symptoms^2,3^. GABA supplements with mental health claims and other promising outcomes are available on the market in some parts of the world, yet it’s uncertain if dietary GABA can cross the Blood Brain Barrier due to conflicting study results and varying research designs^4–6^. Some evidence suggests that GABA’s effects might be instead mediated through the gut–brain axis, particularly via the enteric nervous system and afferent nerves such as the vagus nerve^2,6^. This is also suggested by animal experiments in which the effects on neurochemistry and behaviour were not observed after the vagotomy of the test animals^3^. Besides GABA’s known functions in the brain, it plays an excitatory and inhibitory role in the gut, where it influences secretion and motility, and is involved in the regulation of peptide hormones and neurotransmitters, making it a potential target for treating intestinal diseases^7^, further emphasising its relevance within the gut–brain axis.

**Fig. 1:**
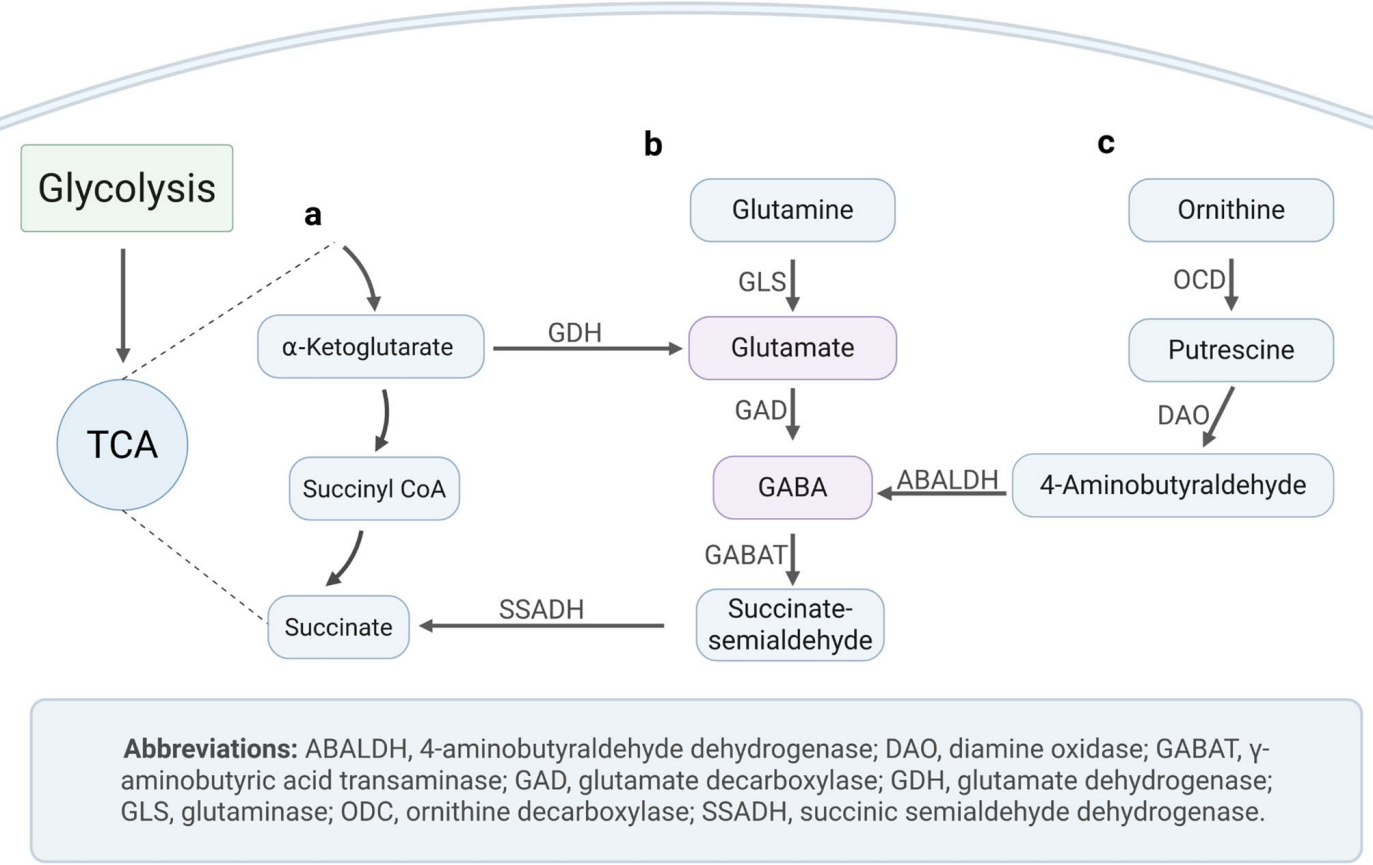
GABA metabolism^7,15,16^. Two known pathways of GABA synthesis in bacteria. **a**) Is showing the citric acid cycle (TCA) **b**) the GABA shunt and **c**) the polyamine pathway. Figure created in BioRender. Kaleta, C. (2026) https://BioRender.com/e1rkjzk.

Bifidobacteria are widely known for their health-beneficial properties^8–10^. According to a previous work, *B. adolescentis* HD17T2H isolated from human faecal samples was found to be one of the highest GABA-producing strains among 82 *B. adolescentis* isolates tested in vitro following a genome-based screening of over 1,000 human gut bacterial reference genomes^11^. GABA is known to be synthesised from L-glutamate via the enzyme glutamate decarboxylase in the GABA shunt. Furthermore, GABA can be synthesised from the polyamine putrescine by diamine oxidase and aldehyde dehydrogenase^7^ (Fig. 1). Several in silico and in vitro studies have shown that among all gut bacteria, lactic acid bacteria like bifidobacteria can produce GABA^7,11,12^. Even though *Bifidobacterium* strains are commonly used as probiotics^13,14^, their metabolism and physiology are not yet fully understood.

The industrial production of probiotics and postbiotics (i.e. metabolic byproducts secreted by bacteria) widely relies on empirical approaches; however, there is a growing need for more systematic, knowledge-based methods to determine the critical nutritional needs of microorganisms^17^. Constraint-based modelling allows us to understand and in silico predict the metabolic behaviour of microorganisms. Constraint-based models are reconstructed based on the genome of an organism. Specifically, the genes are matched to known metabolic reactions, which leads to the creation of metabolic networks that can be further used in simulations^18^. This process can be performed manually or automatically accomplished using automated reconstruction tools such as *gapseq* (18), DEMETER^19^ and CarveMe^20^. Subsequently, with the help of tools such as flux balance analysis^21^ and flux variability analysis^22^, the metabolism of the organisms alone or in communities can be in silico explored. Most interestingly, their metabolism could be engineered to improve the production of compounds of interest and to understand the effects of nutritional supplements and drugs in more detail^23–25^.

While it is known that diets influence the microbiome and that certain bacteria produce neuroactive compounds, the novel contribution of this study lies in its in-silico, model-driven analysis of how specific dietary components affect GABA production. Using genome-scale metabolic modelling, we investigated how different dietary patterns influence GABA production by *B. adolescentis* HD17T2H. We identified specific compounds that support GABA production, which showed considerable differences between the different diets. Specifically, we found carbon and nitrogen sources to have the greatest impact on GABA production. To further explore the potential of GABA production in the human gut, we employed human cohort data of the Kiel cohort and detected other GABA-producing bacteria in this community. Our results call for a deeper exploration of the role of GABA in the human gut to potentially improve host health.

## Results

### In silico prediction of the impact of dietary patterns on GABA production with metabolic modelling

**Fig. 2:**
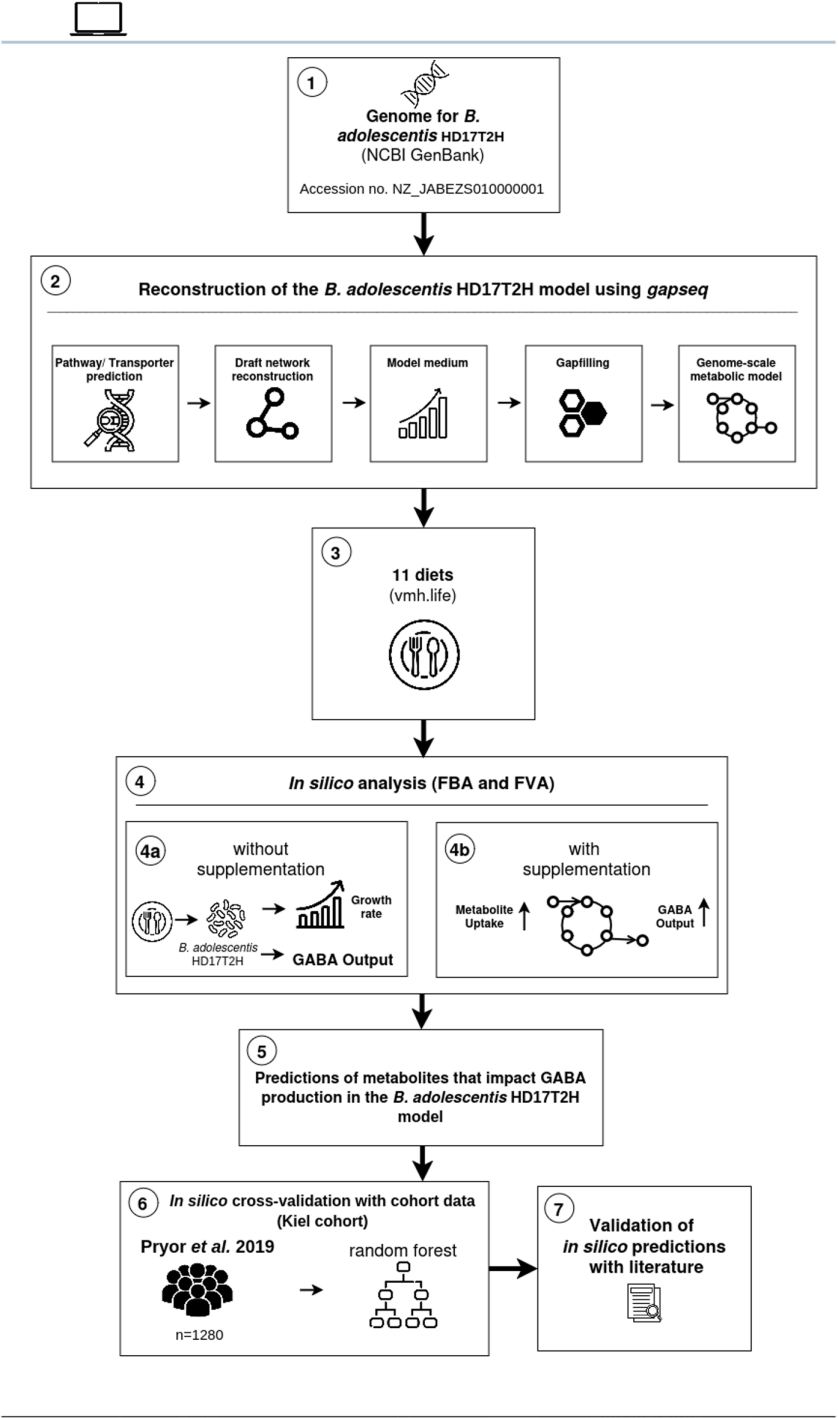
Constraint-based modelling approach to predict the impact of diets on GABA production. (1) The genome data for *B. adolescentis* HD17T2H was taken from the NCBI GenBank (Accession no. NZ_JABEZS010000001). (2) The bacterial model was reconstructed using *gapseq.* 3) 11 diets were taken from the VMH database (www.vmh.life). 4) Flux balance and variability analysis were conducted without (4a) and with (4b) in silico supplementation to predict growth rate, possible GABA output per diet and specific compounds that increase GABA production in the model. 5) In silico predictions of compounds that increase GABA. 6) For in silico cross-validation, dietary inputs of cohort data from the Kiel cohort was used to identify important compounds enhancing GABA production by using a random forest algorithm. 7) In silico predictions were validated with the literature. The graphical abstract was created with draw.io. Icons taken from www.flaticon.com.

To predict the impact of the diets on GABA production capacity of the bacterial model, we performed flux balance analysis to obtain the maximal biomass flux and flux variability analysis to determine the range of possible GABA fluxes. Second, to predict specific compounds that influence GABA production, we compared the GABA production of the model with and without in silico supplementation by using flux balance analysis and flux variability analysis (Fig. 2). By in silico supplementation, we mean that we increased the nutrient uptake in the model to identify which compounds enhance GABA production (Figs. 2 and 4b).

**Fig. 3:**
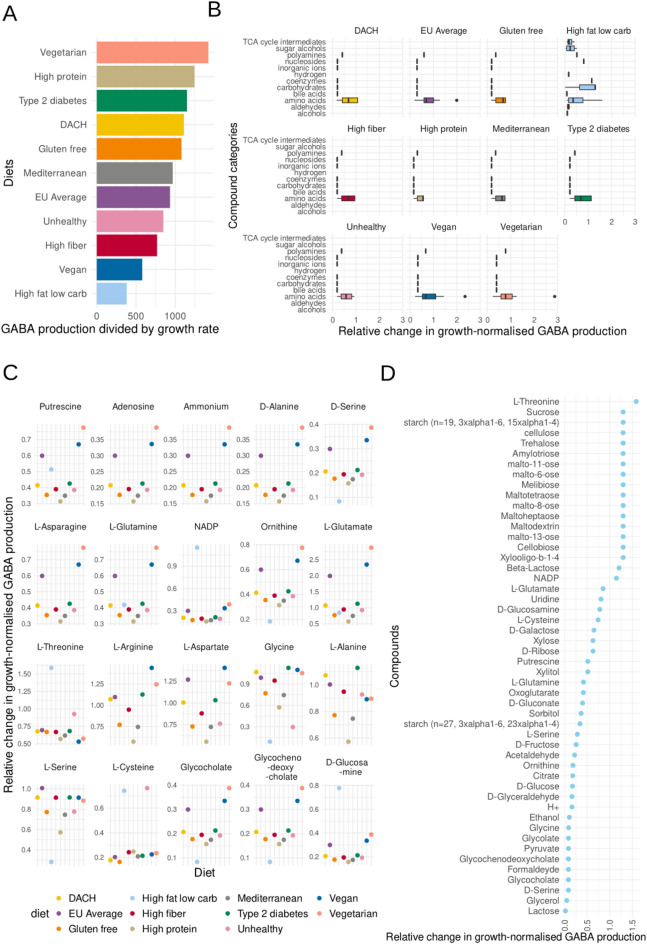
Impact of diets and specific compounds on GABA production in our constraint-based *B. adolescentis* HD17T2H model. **A** Baseline GABA production in mmol/human/day across dietary patterns. GABA production of *B. adolescentis* HD17T2H at baseline without in silico supplementation. The baseline represents the maximal theoretical secretion potential supported by each specific dietary composition, as GABA flux is not mandatory for growth in the model. **B** Effects of diets on GABA production. Relative change in growth-normalised GABA production of *B. adolescentis* HD17T2H on the eleven VMH diets over twelve compound categories after the in silico supplementation, compared to baseline (supplemented − baseline) / baseline). **C** Effects of compounds on GABA production. Zoom-in version of **B** showing the relative change in growth-normalised GABA production at the level of individual supplemented compounds with the strongest influence on GABA production across diets. Using the same metric as in Fig. 3B. **D** Effects of compounds on GABA production after supplementing the high-fat low-carbohydrate diet.

In our simulations, we found noticeable differences in GABA production capacity between the eleven diets. At baseline, i.e. without in silico supplementation, the vegetarian diet showed the highest GABA production capacity, followed by the high protein and type 2 diabetes diet (Fig. 2 (4a), Fig. 3A). The lowest GABA production capacity at baseline was observed for the ketogenic (high-fat low-carbohydrate) diet and the vegan diet (Fig. 3A). The difference between the diet with the highest and the diet with the lowest GABA production capacity at baseline was 73%. To detect relevant compounds for GABA production for each diet, we conducted in silico supplementation experiments by adding compounds into the nutritional input and recording the outcome (Fig. 2 (4b)). After the in silico supplementation, we classified the compounds that we found into 12 different categories (Fig. 3B). In nearly all in silico diets, we found compounds in seven categories that increased GABA production: (1) amino acids, (2) bile acids, (3) carbohydrates, (4) coenzymes, (5) nucleosides, (6) polyamines and (7) inorganic ions (Fig. 3B and C). Most compounds across the categories could be found in the ketogenic diet, with the highest amount in the carbohydrate category, indicating that carbon-rich sources play an important role in GABA production (Fig. 3D). Despite its low baseline GABA levels, the ketogenic diet emerged as the most sensitive to in silico supplementation of carbon sources, showing the greatest potential for increased GABA synthesis upon the addition of key compounds in the model (Fig. 3B and D).

After the in silico supplementation, as expected, L-glutamate, a direct precursor of GABA, was found to be among the most relevant compounds for all of the in silico diets. Moreover, in all eleven diets, putrescine and ornithine were among the important compounds for increased GABA production, which can be explained by GABA being a downstream product of putrescine degradation as part of the polyamine pathway (Figs. 1 and 3C). Further, we detected L-glutamine as a relevant compound to increase GABA production in all eleven virtual diets, which is a known GABA precursor. Further amino acids relevant for GABA production that we identified in the model were L-asparagine, D-alanine, D-serine, L-threonine, L-arginine, L-aspartate, glycine, L-alanine, L-serine and L-cysteine (Fig. 3C). However, in the high-fat low-carbohydrate diet, this effect was not observed for the compounds D-alanine, L-asparagine, L-arginine, L-aspartate, and L-alanine. No effect of L-arginine on GABA production was observed for the high-fat low-carbohydrate and unhealthy diet. Moreover, nicotinamide adenine dinucleotide phosphate (NADP) was identified as a contributor to GABA production, most likely because it is a nitrogen-rich compound which also contains an adenosine moiety. Additionally, adenosine and ammonium were discovered to be relevant in GABA production.

We observed a stronger effect of the in silico supplementation on GABA production in the vegan and vegetarian diets, followed by the average EU diet, for the compounds putrescine, adenosine, ammonium, D-alanine, D-serine, L-asparagine, L-glutamine, ornithine, L-glutamate, glycocholate and glycochenodeoxycholate (Fig. 3C). This suggests that supplementing the identified compounds to these diets helps boost the production of GABA better compared to the other diets.

The highest variation after the in silico supplementation was observed in the low-carbohydrate diet (Fig. 3B and D). The highest rate of change after the in silico supplementation was found for the amino acid L-threonine. Further, we detected a high amount of sugars impacting GABA production in this diet (Fig. 3D). Among these, we found several polysaccharides (e.g. amylotriose and malto-11-ose), disaccharides (e.g. sucrose, trehalose), and monosaccharides (e.g. D-ribose, D-fructose, and D-glucose) (Fig. 3D). Furthermore, we correlated the number of carbon atoms of the compounds we detected in the low-carbohydrate diet with the molecules on GABA production (Fig. S1). As expected, this approach revealed that the more carbon atoms the structure has, the more GABA can be produced (Spearman’s ρ = 0.58, p-value < 0.001 ) (Fig. S1). We further conducted some additional analysis accounting for absorption. Although modelling small-intestinal absorption resulted in lower absolute GABA production levels by the *B. adolescentis* model, the diet-specific effects on GABA production capacity were preserved. Specifically, the relative ranking of all 11 diets was identical between models with and without absorption (Fig. 3A and Fig. S2). Besides, additional analyses were performed to map each supplemented amino acid to the corresponding GABA pathways. Important reactions and KEGG IDs are provided in the supplements (Table T2).

### An alternative metabolic modelling approach for the in silico prediction of dietary compounds that impact GABA production

**Fig. 4:**
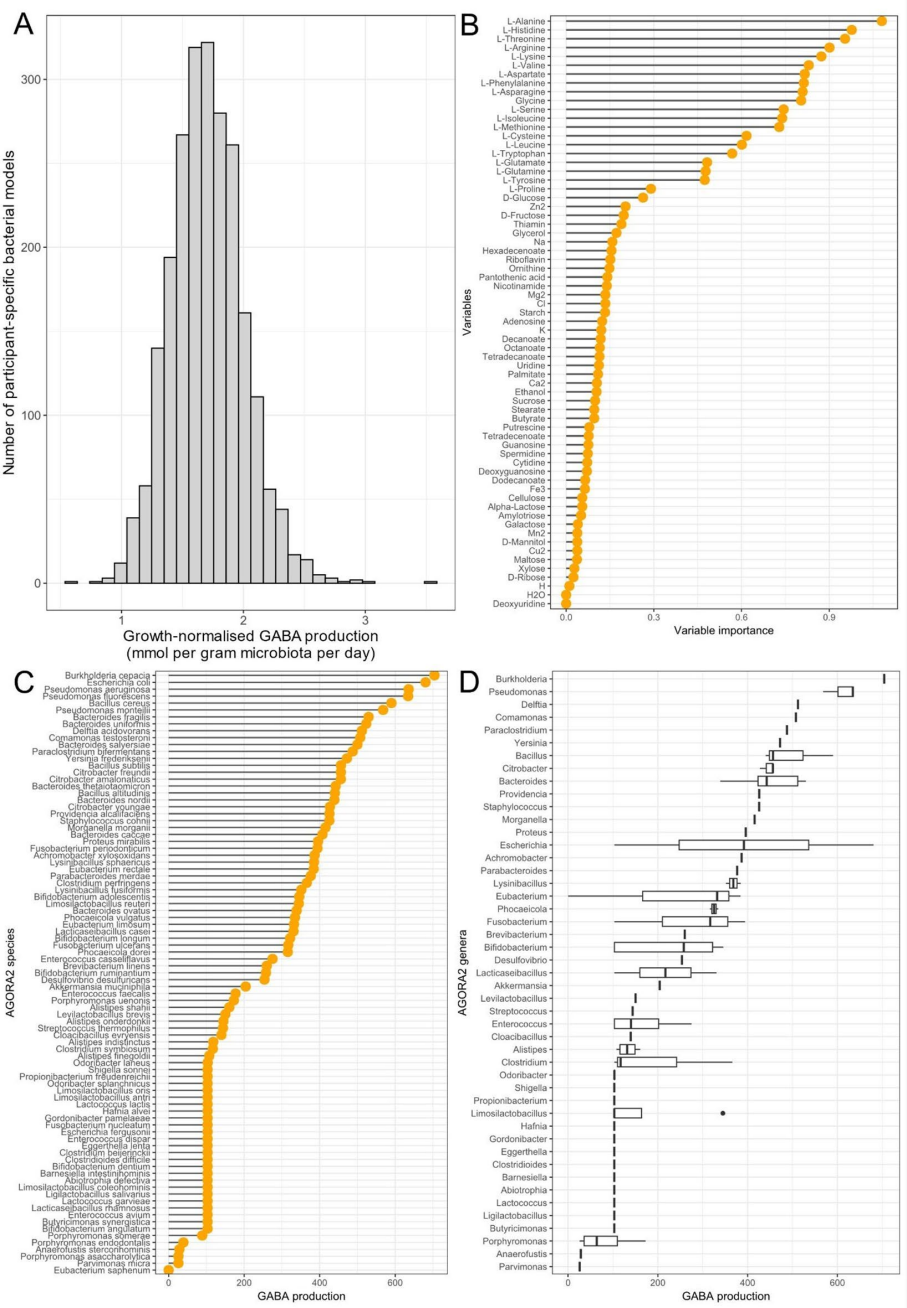
In silico prediction of most important nutritional supplements for GABA production and other GABA-producing bacteria in the Kiel cohort. **A**. Histogram showing the predicted GABA production, normalised by the predicted growth rate of *B. adolescentis*, across the study cohort. The y-axis represents the number of *B. adolescentis* metabolic models achieving the specified production rate, when the molecular information on consumed compounds per participant was used as input for the model (unit: mmol per gram microbiota per day). **B**. Using conditional inference trees, the importance of each compound for the production of GABA was estimated. **C**. Predicted maximum GABA production by AGORA2 species-level models (cutoff: 1st quartile of the distribution; unit: millimoles per human per day) **D**. Predicted maximum GABA production by AGORA2 species-level models agglomerated by genus (unit: millimoles per human per day).

To further investigate the role of specific compounds on the GABA production capacity of *B. adolescentis* HD17T2H, we utilised the molecular information for nutritional compounds consumed by the participants of the Kiel cohort^24,26,27^ and calculated the GABA production for each subject (i.e., maximum FVA production normalised by the achieved biomass) based on FVA. The minimum achieved GABA production was 0.59 mol per gram microbiota per day (mmol/gM/d), the median was 1.70 mmol/gM/d, and the maximum value was 3.54 mmol/gM/d (Fig. 4A). Therefore, we investigated which compounds might be responsible for this observation. To this end, we used a random forest model predicting GABA production from dietary composition and identified variables (i.e. compounds) important for prediction based on the root mean square of the predicted values in comparison to the permuted ones. This in silico analysis revealed that amino acids are by far the most important group for GABA production, followed by other compounds that belong to various groups (e.g., other nitrogen-containing compounds, carbohydrates, vitamins, ions, and fatty acids) (Fig. 4B).

### In silico prediction of GABA-producing species from human cohort data using metabolic modelling

In addition to *B. adolescentis* HD17T2H modelling, we reconstructed the personalised bacterial community of all subjects selected from the Kiel cohort data^24,26^ based on the gut microbiota model collection AGORA2^28^. As explained in the methods, we utilised those community models to identify the GABA producers among the community. Based on the information that 87 bacterial models produced GABA in the personalised bacterial communities, we predicted the individual maximal GABA production based on FVA (Fig. 4C). The most prominent GABA-producing species belonged to 47 genera. The genera *Bukholderia* and *Pseudomonas* were able to produce the highest amounts of GABA, followed by *Delftia* (Fig. 4D). It should be noted that *Bukholderia* is only represented by *Burkholderia cepacia*, which also used to belong to the genus *Pseudomonas*^29^. The maximum production of GABA was 704.11 millimoles per human per day, while the minimum production was 25.88 millimoles per human per day. The mean was 274.74 millimoles per human per day, and the median was 258.79 millimoles per human per day.

## Discussion

Bifidobacteria strains require sugar sources for ideal growth, although differences in carbohydrate degradation pathways exist between strains^30–32^. Our in silico results align with this knowledge and show that sugar sources are important for the production of GABA. This link can be well explained by GABA metabolism since a possible pathway for GABA synthesis is through the GABA shunt which branches off from the TCA cycle and is fed by glycolysis^7,15^ (Fig. 1). Using in silico supplementation experiments, it was revealed that specific monosaccharides (fructose, glucose, ribose, xylose), disaccharides and polysaccharides support GABA synthesis (Fig. 3D). Importantly, the ketogenic diet, characterised by low carbohydrate content, showed the lowest baseline GABA levels but the highest sensitivity to in silico supplementation with carbon sources. Among all diets in the VMH database (www.vmh.life/#nutrition*)*, the vegetarian and vegan diets contain the highest percentage of carbohydrates relative to total energy intake. Both diets are especially sensitive to in silico supplementation of nitrogen sources, as these were the limiting factors in the diets (Fig. 3A–C). This means that the nutrient limiting GABA production varies with diet: in low-carbohydrate diets carbon is the main limitation, while in high-carbohydrate diets nitrogen becomes limiting. Our results suggest that high sugar intake can increase GABA production. However, high Glycemic Index /Glycemic Load (high-GI/GL) diets are linked to increased disease risk^33–35^. Low GABA levels have been found in individuals with depression^36^, and high sugar consumption is associated with depression^33^. Thus, increasing GABA via high-GI/GL diets is not advisable. While our findings improve understanding of bacterial GABA metabolism, they do not inform on diet-disease associations.

The importance of nitrogen for GABA production is well known. In industry, nitrogen sources are used in the fermentation media of microorganisms for targeted GABA synthesis^12,37^. In our in silico model, we found nitrogen-rich compounds, mainly amino acids, to be essential nutrients for GABA production by *B. adolescentis* HD17T2H (Figs. 3C and 4B). According to the random forest model, the first twenty most important compounds for GABA production are amino acids. Interestingly, we found all nine amino acids that cannot be synthesised by humans and (i.e. essential amino acids) therefore are dietary essential to be among the top relevant compounds for GABA production of our *Bifidobacterium* model (Fig. 4B). Therefore, it is not surprising that the high-protein diet was found to have the highest baseline GABA production (Fig. 3A). These results might explain why protein intake is associated with a reduced risk of depression, while low-protein diets are linked with depressive symptoms^38,39^. Further work suggests that the quality and source of protein are also important^40,41^. For instance, a high intake of animal protein has been linked to depressive symptoms, whereas no such association has been found for plant-based proteins^41^.

Apart from virtual dietary patterns, such as the ones of VMH, we employed the Kiel cohort data^26^ containing the molecular information for nutritional compounds for each participant. Comparing the maximum and minimal GABA production values by *B. adolescentis* HD17T2H, our in silico analyses suggest that there is a wide distribution of potential GABA production (Fig. 4A), which depends only on the different personalised, nutritional inputs. As the relationship between diet and microbial output is complex and high-dimensional, we employed a random forest to interpret it^42^. We detected the most important food components influencing GABA production in our subjects (Fig. 4B). As expected, this approach detected nitrogen-rich compounds, most importantly amino acids, to be the main components for increasing GABA production, which is in line with the previous analyses (Fig. 3A and B). A potential metabolic explanation for the importance of alanine and arginine lies in the network structure of the model. For instance, the model contains the reaction L-alanine:2-oxoglutarate aminotransferase (KEGG reaction: R00258), which converts alanine and 2-oxoglutarate to glutamate and pyruvate, and L-arginine amidinohydrolase (KEGG reaction: R00551), which converts arginine to ornithine. While the presence of these reactions provides a theoretical basis for our findings, further analysis of their specific flux would be required to confirm if they are active in our model and directly contribute to the predicted GABA production. Furthermore, comparing those results with the outcomes of the VMH diets (Fig. 3), the analysis using Kiel cohort data showed similar behaviour. In vitro studies found various nitrogen sources, such as methionine or monosodium glutamate, to increase the GABA production of microorganisms^43–45^.

Beyond carbon and nitrogen compounds, our results from the analysis of the Kiel cohort data demonstrate that several fatty acids influence GABA biosynthesis. Specifically, in silico supplementation with hexadecenoate (C16:1), tetradecenoate (C14:1), decanoate (C10:0), octanoate (C8:0), tetradecanoate (C14:0), and dodecanoate (C12:0) increased GABA production by *B. adolescentis* HD17T2H. This highlights a metabolic link between lipid metabolism and microbial neurotransmitter output, which aligns with previous findings^36,37^. This suggests that fatty acids can indirectly fuel neurotransmitter production via the TCA cycle and glutamine metabolism. Taken together, our findings suggest that specific dietary fatty acids not only act as energy sources but may actively promote GABA biosynthesis in *B. adolescentis* HD17T2H. Whether this occurs through direct metabolic pathways or via modulation of redox state, energy status, or cofactor availability remains to be clarified.

Furthermore, our cross-validation analysis of the Kiel cohort data identified specific B vitamins as relevant contributors to GABA synthesis. We found that thiamine (B1), riboflavin (B2), pantothenic acid (B5), and nicotinamide (the amide form of B3) enhanced GABA production in *B. adolescentis* HD17T2H. Although these vitamins act as cofactors and are not used up during GABA synthesis, their presence likely creates metabolic conditions that support GABA production. Their role as cofactors in key TCA cycle enzymes, such as pyruvate dehydrogenase, α-ketoglutarate dehydrogenase, and succinate dehydrogenase, may indirectly promote GABA synthesis by improving flux through the TCA cycle and the connected GABA shunt^47^. In addition to the B vitamins identified through our cohort analysis, the role of pyridoxal-5′-phosphate (PLP), the active form of vitamin B6, merits particular attention as it is the essential cofactor for glutamate decarboxylase (GAD). GAD is the primary enzyme responsible for GABA biosynthesis in bifidobacteria^11^. In our constraint-based modelling approach, the availability of such cofactors is implicitly assumed to be non-limiting within the metabolic network. However, it is well-established that PLP availability can significantly modulate GABA flux; studies on lactic acid bacteria and bifidobacteria have demonstrated that increased PLP levels can enhance GAD activity and, consequently, total GABA production^15,48^. Therefore, while our model predicts the metabolic potential in silico, in vivo GABA production may be further influenced by the systemic or dietary availability of vitamin B6^48^.

An important limitation of our study is that our models did not account for intestinal absorption. While dietary fibres reach the colon, due to their ingestibility, around 92% of dietary amino acids and 99% of simple sugars are absorbed in the small intestine, and only small quantities reach the large intestine^49–51^. Although we did not take absorption into account in the main analysis, our models help to understand the nutritional requirements of *B. adolescentis* HD17T2H better. Besides, the compounds from our diets are not exclusively relevant. Compounds, like amino acids and sugar monomers, can also be derived from the mucus layer of the intestine and be utilised by gut bacteria^52^. In addition, prototrophic bacteria can produce metabolic products from which other bacteria benefit through cross-feeding^53^.

Additionally, the investigation of AGORA2 models (Fig. 4C, D) revealed broad GABA production potential across species and genera. According to our analysis, these species are mostly abundant in the human gut and therefore contribute to microbial GABA production within the gut–brain axis. In addition, we found several GABA-producing bifidobacteria, like *B. adolescentis*, *B. ruminantium*, *B. longum*, *B. angulatum* and *B. dentium*. Nevertheless, in the Kiel cohort data, bifidobacteria seem to play a minor role as GABA producers in the adult human gut, compared to other species (Fig. 4C, D). Moreover, Fig. 4C demonstrates pronounced interspecies variability in GABA production potential within the genus *Bifidobacterium*, for example, between *B. adolescentis* and *B. angulatum.* Furthermore, the ability to produce GABA is not the only reason to name bacteria “protective” or “beneficial”. In our study, for example, *Burkholderia (Pseudomonas) cepacia* appears as a theoretical GABA-producer, although some of its strains are known as opportunistic human pathogens (Fig. 4C)^54^. Similarly, *Delftia acidovorans*, identified in our study as a potential GABA producer, has been implicated in clinical infections. Several case reports have described *D. acidovorans* as the causative agent of catheter-related sepsis, empyema, and bacteremia in both immunocompromised and immunocompetent individuals^55–57^. These examples illustrate that GABA production is widespread and found across diverse taxa, including those not typically regarded as commensal. Our in silico cross-validation using the Kiel cohort shows that numerous bacterial taxa in the adult human gut are GABA-producers (Fig. 4). This is not unexpected. The GABA pathway is common in bacteria because it serves ecologically important functions, including acid stress tolerance and responses to oxidative stress^58,59^. It also supports bacterial survival and growth under oxygen-limiting conditions^58^. Consequently, the presence of GABA-producing taxa in the gut, whether commensal or pathogenic, should be interpreted within a broader physiological and ecological context rather than as an indicator of harm. Overall microbial balance remains the relevant determinant of host impact^60^.

In this study, although metabolic models have proven to be powerful tools to predict metabolic interactions of microorganisms, constraint-based metabolic modelling cannot include non-metabolic features (e.g., gene regulation), and therefore it is important to validate the results of the models with in vitro data. Further, this study mainly focuses on just one bacterial strain and suggests that including more strains in future research could provide a more comprehensive view of how dietary patterns affect the gut microbiome. It is well established that GABA production varies not only between species but also between strains, for example, among different *B. adolescentis* strains, underlining the importance of strain-level modelling^11^. Additionally, considering nutrient absorption rates in the small intestine could improve the accuracy of models regarding the nutrient supply to intestinal bacteria. Therefore we like to emphasise that our in silico model represents an idealised colon. For future research, we suggest laboratory experiments that examine GABA production by bifidobacteria under conditions mimicking diets associated with high GABA production (e.g., vegetarian) and low GABA production (e.g., low-carbohydrate) to validate our findings in vitro. Despite these limitations, our models were sufficiently effective for the study’s aim, to understand the impact of different dietary compositions on GABA production and identify key compounds. Furthermore, we validated our findings with literature reports, which show in vitro that carbohydrate and nitrogen sources are important for GABA production by bifidobacteria^31,32,43–45^.

In summary, our study shows that the nutrient-limiting factor for GABA production by *B. adolescentis* HD17T2H depends on dietary composition in our in silico model. We found noticeable differences in GABA production of the model between the eleven VMH diets. Moreover, we predicted the most important dietary compounds that impact GABA production, which are carbohydrates and nitrogen-rich sources, depending on the baseline diet. The ketogenic diet showed the lowest baseline GABA levels but the highest sensitivity towards in silico supplementation of carbon sources, whereas vegetarian and vegan diets were nitrogen-limited. Further, we in silico identified GABA-producing species from the Kiel cohort data. Thus, our study contributes to a better understanding of microbial GABA production. While further research is needed to shed more light on the role of gut-derived GABA in gut and brain health, these insights could help advance microbiome-based approaches to improve neurological health and develop new therapeutic strategies in future.

## Methods

### Genome-scale metabolic network reconstruction

The genome-scale metabolic model was reconstructed for the bacterial genome of *B. adolescentis* HD17T2H. The genome data were downloaded in FASTA format from NCBI GenBank (Accession no. NZ_JABEZS010000001). The genome data originated from previous work and were isolated from faecal samples of healthy humans^11^. To reconstruct the microbial metabolic model, *gapseq* version 1.2 was used. *gapseq* is a tool that automatically reconstructs genome-scale metabolic models of microorganisms and provides a gap-filling algorithm^18^. For the medium construction, we used the *gapseq* module for medium prediction and removed oxygen to create the ideal growth conditions for the anaerobic bacterial model. We used the default bitscore cutoff 200. The default gram positive biomass reaction was set as the objective function.

### Preparation of the eleven diets

The diets were taken from the VMH (vmh.life)^61^ database. VMH provides information about eleven predefined diets. These diets are designed for in silico simulations and have been used in this context in previous studies^61,62^. The diets are based on literature and surveys and contain the appropriate amounts for a whole-day meal plan. Information about the quantities of each compound in each diet is provided. In total, 91 compounds per diet are listed. If a compound was not part of a diet, the flux rate was zero. The compounds belong to the following categories: fatty acids, amino acids, carbohydrates, dietary fibres, vitamins, minerals, and trace elements. For the metabolic modelling approach, the compound information could be downloaded as flux rates in millimoles per human per day. To prepare the diets for the models, the compound IDs from VMH were translated to those from *gapseq*, which are also based on the ModelSEED IDs. We replaced the predicted medium values generated by *gapseq* for the *B. adolescentis* HD17T2H model with the actual dietary values. As the model did not grow initially, we identified sulphate and cobalt ions as limiting factors and added them to the growth medium. Additionally, we generated a version accounting for the absorption rates in the small intenstine as estimated by Best et al. 2025^63^. More information about the diets can be obtained here^61^. A table containing an overview of the macronutrient compositions (% carbohydrate, fat, protein) of the 11 diets, their key differences, and the references used for diet construction can be found in the supplements (T1). A table with a more detailed description of all 11 diets is available at https://www.vmh.life/#nutrition. To provide full documentation, the complete formulations can be found in the supplementary files together with the utilised original absorption table (Supplementary file in silico diets).

### In silico prediction of the impact on GABA production of the eleven diets

Dietary impact on GABA production was predicted by flux balance analysis and flux variability analysis. Flux balance analysis and flux variability analysis were run for all eleven diets. Flux balance analysis was used to obtain the achieved biomass flux, and flux variability analysis to obtain the range of possible flux that still supports the given optimal functional state by 100 per cent. GABA production rates were normalised by growth rates so that the normalised rates are decoupled from the effect of the diets on the growth rates $(\mathit{GABA\ FVA\ production\ rate/Growth\ rate})$. It should be noted that GABA production is not a requirement for biomass synthesis in our model. Consequently, a standard optimisation for maximal growth alone would result in zero GABA flux. To explore the metabolic capacity of the strain, we used FVA to determine the maximal theoretical secretion potential of GABA while maintaining the optimal growth rate. To identify specific compounds that increase GABA production in the model, a in silico supplementation was done by increasing the lower bounds per diet. Specifically, if the original lower bound of a compound in a diet was less than − 1e-6, then the new lower bound was set to -100 $(new\ lower\ bound = -100)$. Otherwise, the new lower bound was to be set to ten times higher than the original one $(new\ lower\ bound = 10 * old\ lower\ bound)$. Subsequently, flux balance analysis and flux variability analysis were conducted and the maximum flux values for GABA production were extracted. Finally, we explored which internal reactions were driving GABA production by analysing the corresponding flux distribution. Specifically, we forced the model to produce the amount of biomass and GABA observed in the DACH diet and then we analysed the flux distribution generated by the “minimize total flux” optimisation algorithm. The in silico analyses were performed in R version 4.5.1. To run the constraint-based analysis, the R-package *sybil* (Github version SysBioChalmers/sybil@^64^ was used, and cplexAPI v. 1.4.0 (academic licence of IBM ILOG CPLEX 22.1.1.0) to solve the optimisation problems. *Tidyverse* 2.0.0^65^, *grafify* 5.1.0^66^, and *ggpubr* 0.6.1^67^ were chosen for the visualisation.

### In silico prediction of the most relevant dietary compounds for GABA production using cohort data

The Kiel cohort data^26^ hold information on nutritional compounds for each participant and were used to predict dietary compounds that are relevant for GABA production by *B. adolescentis* HD17T2H. This dataset contains the molecular information for nutritional compounds for each participant (mmol/day). The list of participants was so filtered that our subjects were the same subjects that were taken into account in the publication of Pryor et al.., 2019 (*n* = 1280)^24^. Similarly, to the same publication^24^, we divided the nutritional amounts by 200 g to take into account the amount of microbiota in the human colon. The flux unit, therefore, was set to mmol per gram microbiota per day (mmol/gM/d)^24^. To limit artefacts from the dietary input, we added to the medium 0.1 mmol/gM/d phosphate, cobalt, Fe^+ 2^, sulphate, while we excluded from the division by 200 the following compounds: cobalt, manganese, copper and riboflavin, thiamin and pantothenate. Based on this information, we simulated the GABA production capacity of *B. adolescentis* HD17T2H for each subject based on FVA $(\mathit{GABA\ FVA\ production\ rate/Growth\ rate})$. To identify the primary dietary drivers of this metabolic output and following a published approach^42^, we predicted the most relevant compounds by means of random forest (conditional inference trees) using the R library party (version 14)^42,68–72^. The formula used to this end is: $(\mathit{FVA}\ max\ GABA\ flux / the\ achieved\ biomass\ by\ \mathit{FBA}\ \sim\ nutritional\ compounds).$The out-of-bag R-squared for the cforest model was 0.98; the absolute out-of-bag prediction errors for the cforest model were evaluated, with a median absolute error of 0.176. The 25th and 75th percentiles of the absolute errors were 0.085 and 0.314, respectively. The maximum absolute error was 11.602. FBA and FVA were performed using the R-packages *sybil* (Github version SysBioChalmers/sybil@58eb989)^64^ and cplexAPI v. 1.4.0 (academic licence of IBM ILOG CPLEX 22.1.0.0). For plotting, the R packages ggplot2 (version 3.5.1)^73^, flexplot (Github version dustinfife/flexplot@ed65b1f), and forcats (version 1.0.0) were used. The in silico analyses were performed in R version 4.4.1.

### In silico prediction of GABA-producing strains from the human gut

Moving from single-species experiments, we investigated the potential GABA production by species associated with the human gut. To this end, we employed the microbial collection of AGORA2^28^. We restricted our analysis to the list of species present in the Kiel cohort data used before (*n* = 1280)^31^. Specifically, 16 S rRNA counts were aligned to the AGORA2^28^ database using MARS^74^ based on taxonomic names. Adjustments were made to certain names within the dataset to account for changes in taxonomic identity. Subsequently, all reads corresponding to AGORA2-mapped species were normalised per sample, ensuring the resulting sum of relative abundances was equal to 1. Species with abundances below 1e-6 were excluded, and the dataset underwent a final re-normalisation to obtain the ultimate relative abundance values. The Microbiome Modelling Toolbox within the COBRA Toolbox^75^ was employed to generate personalised community models. The EUAverageDietNew, available in the COBRA Toolbox, was applied to constrain the metabolic models. Microbiome biomass growth was restricted to 1. Each of the 1280 personalised community models was then analysed using the *predictMicrobeContributions* function, which maximises the secretion flux through the GABA (VMH id: 4abut) exchange reaction of each bacterial model within the respective community. Taking into consideration all personalised bacterial communities, 87 community members produced GABA. Since the production of GABA strongly correlated to the bacterial abundances, which indicates that it reached the bounds imposed by the coupling constraints, we used the species-level AGORA2 models corresponding to the 87 aforementioned species to simulate the individual microbial production potentials of GABA using FVA. The in silico reconstructions and predictions were performed in MATLAB (version 2021b).

## Data Availability

The computer code and the simulation results are available in Figshare: In silico diets from VMH.life: DOI: 10.6084/m9.figshare.26187119. Figures: DOI: 10.6084/m9.figshare.26187377. Scripts: DOI: 10.6084/m9.figshare.26187533. Bacterial model: DOI: 10.6084/m9.figshare.26187095. Supplementary Files: DOI: 10.6084/m9.figshare.30788639. The Kiel cohort data used in this study is accessible through an online structured application (https://www.uksh.de/P2N/) to the PopGen 2.0 Network.

## Abbreviations

FBA: Flux balance analysis
FVA: Flux variability analysis
VMH: Virtual metabolic human
GABA: Gamma-aminobutyric acid
SCFA: Short chain fatty acids

## Declarations

### Compliance with guidelines

All methods were carried out in accordance with relevant guidelines and regulations.

## References

[1] Petroff, O. A. C. Book Review: GABA and Glutamate in the Human Brain. *Neuroscientist* **8** (6), 562–573 (2002). 12467378 10.1177/1073858402238515

[2] Cawthon, C. R. & De La Serre, C. B. Gut bacteria interaction with vagal afferents. *Brain Res.* **1693**, 134–139 (2018). 29360469 10.1016/j.brainres.2018.01.012

[3] Bravo, J. A. et al. Ingestion of Lactobacillus strain regulates emotional behavior and central GABA receptor expression in a mouse via the vagus nerve. *Proc. Natl. Acad. Sci.* **108** (38), 16050–16055 (2011). 21876150 10.1073/pnas.1102999108 PMC3179073

[4] Kuriyama, K. & Sze, P. Y. Blood-brain barrier to H3-gamma-aminobutyric acid in normal and amino oxyacetic acid-treated animals. *Neuropharmacology*, **10**(1), 103-108 (1971). 10.1016/0028-3908(71)90013-x 5569303

[5] Gitte Moos Knudsen, I. Henrik Enghusen Poulsen 2* and Olaf B. Paulson I. Blood-brain barrier permeability in galactosamine-induced hepatic encephalopathy: no evidence for increased GABA-transport. **Journal of hepatology**, **6**(2), 187-192 (1988). 10.1016/s0168-8278(88)80030-8 3411098

[6] Boonstra, E. et al. Neurotransmitters as food supplements: the effects of GABA on brain and behavior. Front Psychol [Internet]. Oct 6 [cited 2024 Feb 8];6. Available from: http://journal.frontiersin.org/Article/ (2015). 10.3389/fpsyg.2015.01520/abstract 10.3389/fpsyg.2015.01520 PMC4594160 26500584

[7] Krantis, A. GABA in the Mammalian Enteric Nervous System. *Physiology* **15** (6), 284–290 (2000). 10.1152/physiologyonline.2000.15.6.284 11390928

[8] Ku, S. et al. The role of Bifidobacterium in longevity and the future of probiotics. *Food Sci. Biotechnol.* **33** (9), 2097–2110 (2024). 39130652 10.1007/s10068-024-01631-y PMC11315853

[9] Chen, S. et al. Bifidobacterium adolescentis regulates catalase activity and host metabolism and improves healthspan and lifespan in multiple species. *Nat. Aging*. **1** (11), 991–1001 (2021). 37118342 10.1038/s43587-021-00129-0

[10] Patterson, E. et al. Bifidobacterium longum 1714 improves sleep quality and aspects of well-being in healthy adults: a randomized, double-blind, placebo-controlled clinical trial. *Sci. Rep.* **14** (1), 3725 (2024). 38355674 10.1038/s41598-024-53810-w PMC10866977

[11] Duranti, S. et al. Bifidobacterium adolescentis as a key member of the human gut microbiota in the production of GABA. *Sci. Rep.* **10** (1), 14112 (2020). 32839473 10.1038/s41598-020-70986-z PMC7445748

[12] Li, H., Qiu, T., Gao, D. & Cao, Y. Medium optimization for production of gamma-aminobutyric acid by Lactobacillus brevis NCL912. *Amino Acids*. **38** (5), 1439–1445 (2010). 19787432 10.1007/s00726-009-0355-3

[13] Hidalgo-Cantabrana, C. et al. Bifidobacteria and Their Health-Promoting Effects. *Microbiol Spectr*. 5(3):5.3.21. (2017). 10.1128/microbiolspec.bad-0010-2016 PMC11687494 28643627

[14] Biavati, B., Vescovo, M., Torriani, S. & Bottazzi, V. Bifidobacteria: history, ecology, physiology and applications. *Ann. Microbiol.* **50** (2), 117–131 (2000).

[15] Pannerchelvan, S. et al. Strategies for improvement of gamma-aminobutyric acid (GABA) biosynthesis via lactic acid bacteria (LAB) fermentation. *Food Funct.* **14** (9), 3929–3948 (2023). 36951915 10.1039/d2fo03936b

[16] Zhang, C. et al. Research progress on the microbial metabolism and transport of polyamines and their roles in animal gut homeostasis. *J. Anim. Sci. Biotechnol.* **16** (1), 57 (2025). 40234982 10.1186/s40104-025-01193-x PMC11998418

[17] Rau, M. H. & Zeidan, A. A. Constraint-based modeling in microbial food biotechnology. *Biochem. Soc. Trans.* **46** (2), 249–260 (2018). 29588387 10.1042/BST20170268 PMC5906707

[18] Zimmermann, J., Kaleta, C. & Waschina, S. gapseq: informed prediction of bacterial metabolic pathways and reconstruction of accurate metabolic models. *Genome Biol.* **22** (1), 81 (2021). 33691770 10.1186/s13059-021-02295-1 PMC7949252

[19] Heinken, A., Magnúsdóttir, S., Fleming, R. M. T. & Thiele, I. DEMETER: efficient simultaneous curation of genome-scale reconstructions guided by experimental data and refined gene annotations. *Bioinformatics* **37** (21), 3974–3975 (2021). 34473240 10.1093/bioinformatics/btab622 PMC8570805

[20] Machado, D., Andrejev, S., Tramontano, M. & Patil, K. R. Fast automated reconstruction of genome-scale metabolic models for microbial species and communities. *Nucleic Acids Res.* **46** (15), 7542–7553 (2018). 30192979 10.1093/nar/gky537 PMC6125623

[21] Antoniewicz, M. R. A guide to metabolic flux analysis in metabolic engineering: Methods, tools and applications. *Metab. Eng.* **63**, 2–12 (2021). 33157225 10.1016/j.ymben.2020.11.002

[22] Kenefake, D., Armingol, E., Lewis, N. E. & Pistikopoulos, E. N. An improved algorithm for flux variability analysis. *BMC Bioinform.* **23** (1), 550 (2022). 10.1186/s12859-022-05089-9 PMC9761963 36536290

[23] Raman, K. & Chandra, N. Flux balance analysis of biological systems: applications and challenges. *Brief. Bioinform*. **10** (4), 435–449 (2009). 19287049 10.1093/bib/bbp011

[24] Pryor, R. et al. Host-Microbe-Drug-Nutrient Screen Identifies Bacterial Effectors of Metformin Therapy. *Cell* **178** (6), 1299–1312e29 (2019). 31474368 10.1016/j.cell.2019.08.003 PMC6736778

[25] Marinos, G. et al. Metabolic model predictions enable targeted microbiome manipulation through precision prebiotics. *Microbiol. Spectr.* **12** (2), e01144–e01123 (2024). 38230938 10.1128/spectrum.01144-23 PMC10846184

[26] Van Dam, E. et al. Sugar-Induced Obesity and Insulin Resistance Are Uncoupled from Shortened Survival in Drosophila. *Cell. Metab.* **31** (4), 710–725e7 (2020). 32197072 10.1016/j.cmet.2020.02.016 PMC7156915

[27] Geisler, C. et al. Cohort profile: the Food Chain Plus (FoCus) cohort. *Eur. J. Epidemiol.* **37** (10), 1087–1105 (2022). 36245062 10.1007/s10654-022-00924-y PMC9630232

[28] Heinken, A. et al. Genome-scale metabolic reconstruction of 7,302 human microorganisms for personalized medicine. *Nat. Biotechnol.* **41** (9), 1320–1331 (2023). 36658342 10.1038/s41587-022-01628-0 PMC10497413

[29] Yabuuchi, E. et al. Proposal of Burkholderia gen. nov. and Transfer of Seven Species of the Genus Pseudomonas Homology Group II to the New Genus, with the Type Species Burkholderia cepacia (Palleroni and Holmes 1981) comb. nov. *Microbiol. Immunol.* **36** (12), 1251–1275 (1992). 1283774 10.1111/j.1348-0421.1992.tb02129.x

[30] Schöpping, M., Gaspar, P., Neves, A. R., Franzén, C. J. & Zeidan, A. A. Identifying the essential nutritional requirements of the probiotic bacteria Bifidobacterium animalis and Bifidobacterium longum through genome-scale modeling. *Npj Syst. Biol. Appl.* **7** (1), 47 (2021). 34887435 10.1038/s41540-021-00207-4 PMC8660834

[31] Murakami, R. et al. Preferential sugar utilization by bifidobacterial species. Microbiome Res Rep [Internet]. [cited 2025 Dec 3];2(4). Available from: https://www.oaepublish.com/articles/mrr.2023.19 (2023). 10.20517/mrr.2023.19 PMC10688810 38045925

[32] Altaib, H. et al. Cell factory for γ-aminobutyric acid (GABA) production using Bifidobacterium adolescentis. *Microb. Cell. Factories*. **21** (1), 33 (2022). 10.1186/s12934-021-01729-6 PMC8903651 35255900

[33] Rahimlou, M., Morshedzadeh, N., Karimi, S. & Jafarirad, S. Association between dietary glycemic index and glycemic load with depression: a systematic review. *Eur. J. Nutr.* **57** (7), 2333–2340 (2018). 29744611 10.1007/s00394-018-1710-5

[34] Malik, V. S., Pan, A., Willett, W. C. & Hu, F. B. Sugar-sweetened beverages and weight gain in children and adults: a systematic review and meta-analysis. *Am. J. Clin. Nutr.* **98** (4), 1084–1102 (2013). 23966427 10.3945/ajcn.113.058362 PMC3778861

[35] Salmerón, J. et al. Dietary Fiber, Glycemic Load, and Risk of NIDDM in Men. *Diabetes Care*. **20** (4), 545–550 (1997). 9096978 10.2337/diacare.20.4.545

[36] Epperson, C. N. et al. Preliminary evidence of reduced occipital GABA concentrations in puerperal women: a 1H-MRS study. *Psychopharmacol. (Berl)*. **186** (3), 425–433 (2006). 10.1007/s00213-006-0313-7 16724188

[37] Marlida, Y., Wizna, W., Jamsari, J., Mirzah, M. & Anggraini, L. Optimization of Nutrient Medium for Pediococcus acidilactici DS15 to Produce GABA. *J. Worlds Poult. Res.* **9** (3), 139–146 (2019).

[38] Pooyan, S. et al. A high-protein/low-fat diet may interact with vitamin D-binding protein gene variants to moderate the risk of depression in apparently healthy adults. *Lifestyle Genomics*. **11** (1), 64–72 (2018). 30184533 10.1159/000492497

[39] Lee, D. Y. et al. Association of a low protein diet with depressive symptoms and poor health-related quality of life in CKD. *J. Psychiatr Res.* **161**, 282–288 (2023). 36947959 10.1016/j.jpsychires.2023.02.032

[40] Khanna, P. & Aeri, B. T. Association of Quantity and Quality of Protein Intake with Depression and Anxiety Symptoms among Adolescent Boys and Girls (13–15 Years) Studying in Public Schools of Delhi. *J. Nutr. Sci. Vitaminol (Tokyo)*. **66** (Supplement), S141–S148 (2020). 33612584 10.3177/jnsv.66.S141

[41] Sheikhi, A., Siassi, F., Djazayery, A., Guilani, B. & Azadbakht, L. Plant and animal protein intake and its association with depression, anxiety, and stress among Iranian women. *BMC Public. Health*. **23** (1), 161 (2023). 36694166 10.1186/s12889-023-15100-4 PMC9872399

[42] Fife, D. A. & D’Onofrio, J. Common, uncommon, and novel applications of random forest in psychological research. *Behav. Res. Methods*. **55** (5), 2447–2466 (2022). 35915361 10.3758/s13428-022-01901-9

[43] Wang, J. J., Lee, C. L. & Pan, T. M. Improvement of monacolin K, y-aminobutyric acid and citrinin production ratio as a function of environmental conditions of Monascus purpureus NTU 601. *J. Ind. Microbiol. Biotechnol.* **30** (11), 669–676 (2003). 14625794 10.1007/s10295-003-0097-2

[44] Villegas, J. M., Brown, L., Savoy De Giori, G. & Hebert, E. M. Optimization of batch culture conditions for GABA production by Lactobacillus brevis CRL 1942, isolated from quinoa sourdough. *LWT - Food Sci. Technol.* **67**, 22–26 (2016).

[45] Rayavarapu, B., Tallapragada, P. & Ms, U. Optimization and comparison of ℽ-aminobutyric acid (GABA) production by LAB in soymilk using RSM and ANN models. *Beni-Suef Univ. J. Basic. Appl. Sci.* **10** (1), 14 (2021).

[46] Andersen, J. V. et al. Astrocyte metabolism of the medium-chain fatty acids octanoic acid and decanoic acid promotes GABA synthesis in neurons via elevated glutamine supply. *Mol. Brain*. **14**(1), 132 (2021). 34479615 10.1186/s13041-021-00842-2 PMC8414667

[47] Depeint, F., Bruce, W. R., Shangari, N., Mehta, R. & O’Brien, P. J. Mitochondrial function and toxicity: Role of the B vitamin family on mitochondrial energy metabolism. *Chem. Biol. Interact.* **163** (1–2), 94–112 (2006). 16765926 10.1016/j.cbi.2006.04.014

[48] Li, H. et al. Production of Gamma-Aminobutyric Acid by Levilactobacillus brevis CD0817 by Coupling Fermentation with Self-Buffered Whole-Cell Catalysis. *Fermentation* **8** (7), 321 (2022).

[49] Chambers, E. S., Preston, T., Frost, G. & Morrison, D. J. Role of Gut Microbiota-Generated Short-Chain Fatty Acids in Metabolic and Cardiovascular Health. *Curr. Nutr. Rep.* **7** (4), 198–206 (2018). 30264354 10.1007/s13668-018-0248-8 PMC6244749

[50] Elmadfa, I. *Ernährung des Menschen* 5th edn, 788 (UTB, 2015).

[51] Sareen, S., Gropper, J. L. & Smith *Advanced Nutrition and Human Metabolism* 5th edn, 624 (Cengage Learning, 2008).

[52] Ottman, N. et al. Genome-Scale Model and Omics Analysis of Metabolic Capacities of Akkermansia muciniphila Reveal a Preferential Mucin-Degrading Lifestyle. *Appl. Environ. Microbiol.* **83** (18), e01014–e01017 (2017). 28687644 10.1128/AEM.01014-17 PMC5583483

[53] Giri, S., Yousif, G., Shitut, S., Oña, L. & Kost, C. Prevalent emergence of reciprocity among cross-feeding bacteria. *ISME Commun.* **2** (1), 71 (2022). 37938764 10.1038/s43705-022-00155-y PMC9723789

[54] LiPuma, J. J. Burkholderia cepacia complex as human pathogens. *J. Nematol*. **35** (2), 212 (2003). 19265997 PMC2620619

[55] Lang, K. J., Chinzowu, T. & Cann, K. J. Delftia acidovorans as an Unusual Causative Organism in Line-Related Sepsis. *Indian J. Microbiol.* **52** (1), 102–103 (2012). 23450157 10.1007/s12088-011-0221-3 PMC3298582

[56] Khan, S., Sistla, S., Dhodapkar, R. & Parija, S. C. Fatal Delftia acidovorans infection in an immunocompetent patient with empyema. *Asian Pac. J. Trop. Biomed.* **2** (11), 923–924 (2012). 23569872 10.1016/S2221-1691(12)60254-8 PMC3609244

[57] Kawamura, I. et al. Recurrent vascular catheter-related bacteremia caused by Delftia acidovorans with different antimicrobial susceptibility profiles. *J. Infect. Chemother.* **17** (1), 111–113 (2011). 20628778 10.1007/s10156-010-0089-x

[58] Rahman, A. ur. Studies in natural products chemistry. Amsterdam: Elsevier; (Studies in natural products chemistry). (2018).

[59] Otaru, N. et al. GABA Production by Human Intestinal Bacteroides spp.: Prevalence, Regulation, and Role in Acid Stress Tolerance. *Front. Microbiol.* **12**, 656895 (2021). 33936013 10.3389/fmicb.2021.656895 PMC8082179

[60] Van Hul, M. et al. What defines a healthy gut microbiome? *Gut* **73** (11), 1893–1908 (2024). 39322314 10.1136/gutjnl-2024-333378 PMC11503168

[61] Noronha, A. et al. The Virtual Metabolic Human database: integrating human and gut microbiome metabolism with nutrition and disease. *Nucleic Acids Res.* **47** (D1), D614–D624 (2019). 30371894 10.1093/nar/gky992 PMC6323901

[62] Alessi, D. S., McCreery, C. V. & Zomorrodi, A. R. In silico dietary interventions using whole-body metabolic models reveal sex-specific and differential dietary risk profiles for metabolic syndrome. *Front. Physiol.* **16**, 1586750 (2025). 40470351 10.3389/fphys.2025.1586750 PMC12133891

[63] Best, L. et al. Metabolic modelling reveals the aging-associated decline of host–microbiome metabolic interactions in mice. *Nat. Microbiol.* **10** (4), 973–991 (2025). 40140706 10.1038/s41564-025-01959-z PMC11964932

[64] Gelius-Dietrich, G., Desouki, A. A., Fritzemeier, C. J. & Lercher, M. J. sybil – Efficient constraint-based modelling in R. *BMC Syst. Biol.* **7** (1), 125 (2013). 24224957 10.1186/1752-0509-7-125 PMC3843580

[65] Wickham, H. et al. Welcome to the Tidyverse. *J. Open. Source Softw.* **4** (43), 1686 (2019).

[66] Shenoy, A. R. grafify: an R package for easy graphs, ANOVAs and post-hoc comparisons. Zenodo [Internet]. Available from: 10.5281/zenodo.5136508 (2021).

[67] Kassambara, A. & ggpubr ‘ggplot2’ Based Publication Ready Plots. CRAN [Internet]. Available from: https://rpkgs.datanovia.com/ggpubr/ (2023).

[68] Hothorn, T., Hornik, K. & Zeileis, A. Unbiased Recursive Partitioning: A Conditional Inference Framework. *J. Comput. Graph Stat.* **15** (3), 651–674 (2006).

[69] Zeileis, A., Hothorn, T. & Hornik, K. Model-Based Recursive Partitioning. *J. Comput. Graph Stat.* **17** (2), 492–514 (2008).

[70] Hothorn, T. Survival ensembles. *Biostatistics* **7** (3), 355–373 (2005). 16344280 10.1093/biostatistics/kxj011

[71] Strobl, C., Boulesteix, A. L., Zeileis, A. & Hothorn, T. Bias in random forest variable importance measures: Illustrations, sources and a solution. *BMC Bioinform.* **25**(1), (2007). 10.1186/1471-2105-8-25 PMC1796903 17254353

[72] Strobl, C., Boulesteix, A. L., Kneib, T., Augustin, T. & Zeileis, A. Conditional variable importance for random forests. *BMC Bioinform.* **9** (1), 307 (2008). 10.1186/1471-2105-9-307 PMC2491635 18620558

[73] Wickham, H. ggplot2: Elegant Graphics for Data Analysis [Internet]. Springer-Verlag New York; Available from: https://ggplot2.tidyverse.org (2016).

[74] Hulshof, T., Nap, B., Martinelli, F. & Thiele, I. Microbial Abundances Retrieved from Sequencing data—automated NCBI Taxonomy (MARS): a pipeline to create relative microbial abundance data for the Microbiome Modelling Toolbox and utilizing homosynonyms for efficient mapping to resources. *Bioinforma Adv.* **4** (1), vbae068 (2024). 10.1093/bioadv/vbae068 PMC11193099 38911823

[75] Heinken, A. & Thiele, I. Microbiome Modelling Toolbox 2.0: efficient, tractable modelling of microbiome communities. *Bioinformatics* **38** (8), 2367–2368 (2022). 35157025 10.1093/bioinformatics/btac082 PMC9004645

